# Supplementary material for: Identification of photoperiod-induced specific miRNAs in the adrenal glands of Sunite sheep (Ovis aries)
Source: Front Vet Sci. 2022 Jul 22;9:888207. doi: 10.3389/fvets.2022.888207 (PMC9354845; doi:10.3389/fvets.2022.888207)
Supplement: Supplementary file 1 [file Data_Sheet_1.ZIP › Supplementary file/miRDeep2 file.zip!/miRDeep2 file/Novel_Structure/LP42A3/Novel_255.pdf]

Provisional ID	:	NC_019468.2_13736
Score total	:	1.5
Score for star read(s)	:	-1.3
Score for read counts	:	0
Score for mfe	:	1.2
Score for randfold	:	1.6
Score for cons. seed	:	
Total read count	:	126
Mature read count	:	126
Loop read count	:	0
Star read count	:	0

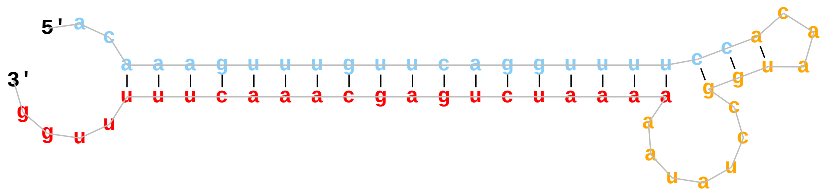

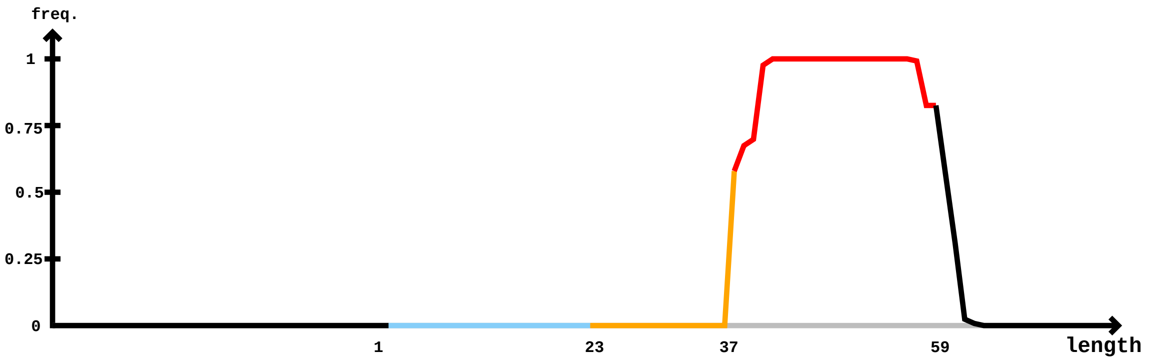

Star Mature

```
5'- ggagaucgagagcagaaaaauacugggcuggccacaaaguuuguucagguuuuccacaauggccuauaaaaaaucugagcaaacuuuuuggccaacacaauaaaaggac   -3'    exp
    ........((..(((.......)))..))(((((.(((((((((((((((((((((...))).......)))))))))))))))))).))))))................    reads     mm         sample
    .....................................................................aaaaCcugagcaaacuuuu.....................    1         1          seq
    .....................................................................aaaaucugaAcaaacuuuuu....................    20        1          seq
    .....................................................................aaaaucugaAcaaacuuuuugg..................    51        1          seq
    .....................................................................aaaaucugaAcaaacuuuuuggc.................    1         1          seq
    ......................................................................aaaucugagcaaacuuuuu....................    1         0          seq
    ......................................................................aaaucugaAcaaacuuuuugg..................    11        1          seq
    .......................................................................aaucugaAcaaacuuuuuggc.................    1         1          seq
    .......................................................................aaucugaAcaaacuuuuuggcc................    2         1          seq
    ........................................................................aucugaAcaaacuuuuuggcc................    34        1          seq
    ........................................................................aucugaAcaaacuuuuuggccaa..............    1         1          seq
    .........................................................................ucugaAcaaacuuuuuggc.................    1         1          seq
    .........................................................................ucugaAcaaacuuuuuggcca...............    2         1          seq
```
